# Cancer incidence in the Tobruk area, eastern Libya: first results from Tobruk Medical Centre

**DOI:** 10.4178/epih.e2021050

**Published:** 2021-08-03

**Authors:** Faisal Ismail, Ahmed G. Elsayed, Islam El-Garawani, Eman Abdelsameea

**Affiliations:** 1 Department of Laboratory, Faculty of Medical Technology, University of Tobruk, Tobruk, Libya; 2 National Centre for Disease Control, Tobruk, Libya; 3 Libyan Medical Research Centre, Kambut, Libya; 4 Pathology Department, Tobruk Medical Centre, Tobruk, Libya; 5 Zoology Department, Faculty of Science, Menoufia University, Shebin El-Kom, Egypt; 6 Hepatology and Gastroenterology Department, National Liver Institute, Menoufia University, Shebin El-Kom, Egypt

**Keywords:** Cancer, Malignancy, Tumors, Cancer incidence, Libya, North Africa

## Abstract

**OBJECTIVES:**

Cancer is a major cause of morbidity and mortality worldwide, and it is an increasing problem in developing countries. Estimation of the incidence of cancer is important, especially in regions with limited epidemiological data on cancer. Therefore, the aim of this study was to provide an updated report on the incidence of cancers in the Tobruk region in eastern Libya.

**METHODS:**

Data on cancer patients from the records of the Department of Histopathology of Tobruk Medical Centre from January 2013 to June 2020 were included.

**RESULTS:**

In total, 402 cases were recorded. Men patients accounted for 30.3% (n=122) of cases, and women patients represented 69.6% (n=280). The overall mean age at the time of the first diagnosis was 49.0±17.1 years. The most common malignancies were breast and uterine cancer in women (18.4%, n=74; 15.9%, n=64, respectively), colorectal cancer (11.6%, n=47; 26 in women and 21 in men), bladder cancer (8.2%, n=33; 8 in women and 25 in men), and thyroid cancer (8.0%, n=32; 23 in women and 9 in men).

**CONCLUSIONS:**

Breast and uterine cancers were the most common cancers in women, and bladder and colorectal cancer were the most common cancers in men, followed by colorectal cancer in both genders. These data will help health authorities launch preventive plans for cancer in the region. Further studies to identify aetiological factors and cancer-related risk factors need to be conducted in the region.

## INTRODUCTION

Cancer is one of the leading health problems throughout the world; in 2018, the World Health Organization estimated that cancer was responsible for 9.6 million deaths. The majority of cancer-related deaths occur in developing countries [1].

The rate of cancer incidence is growing in developing countries because of aging and several cancer-associated risk factors, which include smoking, obesity, physical inactivity, and some chronic infections; for instance, hepatitis B virus and hepatitis C virus (HCV) are associated with liver cancer, human papillomavirus is linked to cervical cancer, and *Helicobacter pylori* is associated with stomach cancer [2].

Most developing countries have little data about the rate of cancer incidence [3]. In the Tobruk region in eastern Libya, there is no cancer registry for the collection and management of data on cancer patients. Cancer registries play an important role in planning and performing research on the causes of cancer, and are also useful for evaluating prevention and control programs targeting cancer in the population [4].

Some reports about the cancer incidence in Libya are available, mainly from the Benghazi region and the western region of Libya [5-10]. However, no study has yet been conducted in the Tobruk region.

This study was conducted to provide the first report on cancer incidence based on data from Tobruk Medical Centre in eastern Libya from 2013 to 2020. It is expected that these data will be a guide for health authorities to plan prevention and control policies for cancer in the region.

## MATERIALS AND METHODS

The present study analysed data from the records of patients who were diagnosed at the Department of Histopathology of Tobruk Medical Centre from January 1, 2013 to June 30, 2020. This department received all the cancer cases from the Tobruk region.

The patients were diagnosed using histopathology sample confirmation techniques. The available data were recorded for each patient, including age, gender, and type of cancer.

### Statistical analysis

The data were analysed with IBM SPSS version 20.0 (IBM Corp., Armonk, NY, USA). Patients’ age was presented as mean ± standard deviation. Frequencies and percentages were computed, and comparisons were made using chi-square analysis to examine the significance of relationships between different variables in the data. A p-value less than 0.05 was considered to indicate statistical significance.

### Ethics statement

The study conformed to the ethical guidelines of the 1975 Declaration of Helsinki, and the study plan was approved by the Ethical Committee for scientific research at Tobruk University (No. 12.20.150). Data in this study were retrieved from the records of the Department of Histopathology with the approval of the hospital ethical committee. The demographic and epidemiological data of patients were retrieved anonymously.

## RESULTS

**Figure 1.**
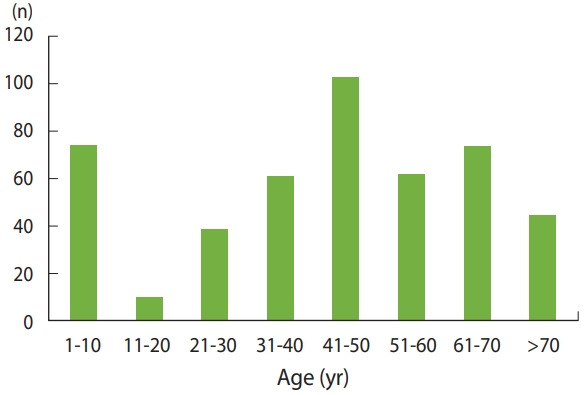
Age distribution and incidence rates of cancers.

A total of 402 cases were recorded at the Department of Histopathology of Tobruk Medical Centre from January 2013 to June 2020. Among them, 31.1% (n= 125) were in men patients and 68.9% (n= 277) were in women patients. The age distribution of patients is presented in Figure 1. The overall average age of the cancer patients at presentation was 49.0± 17.1 years.

The most common malignancies were breast and uterine cancers in women (18.4%, n= 74; 15.9%, n= 64, respectively), colorectal cancer (11.6%, n= 47; 26 in women and 21 in men), bladder cancer (8.2%, n= 33; 8 in women and 25 in men), and thyroid cancer (8.0%, n= 32; 23 in women and 9 in men). The most common malignancies in the study population are listed in Table 1. The majority of cancer cases (n= 284, 70.6%) were in the age groups of 41 years and above.

## DISCUSSION

Studies in the field of cancer epidemiology are limited in developing countries [11], and this is also the case for Tobruk District, which is one of the largest Libyan districts in the east of the country. The Department of Histopathology of Tobruk Medical Centre is the only histopathology department in the region. No study has previously investigated cancer incidence in the Tobruk region. This study evaluated the rate of cancer incidence in the region over the study period from 2013 to 2020.

Several studies on cancer incidence have been carried out other regions of Libya in recent years [3,7,10,12-19]. However, it is important to determine whether there are differences in cancer rates in various areas of the country.

The most common malignancies in our study were breast cancer in women (18.4%, n= 74); this rate of breast cancer is comparable to previous reports from other regions of Libya [7,12,16,17,20] and lower than that reported in other studies elsewhere [21]. Breast cancer is one of the most common cancers worldwide, and the implementation of an effective early detection program is essential to detect cases at an early stage, when there is a higher likelihood of cure and recovery [22].

The second most common cancer in both genders was colorectal cancer; some studies from Libya reported a percentage of colorectal cancer similar to our study in both men and women [6,7].

In neighbouring Egypt, the commonest malignancy in both genders was hepatocellular carcinoma (HCC) (23.8%), followed by breast (15.4%), and bladder cancer (6.9%) [20]. In our study, the histopathology records did not contain any data for HCC over the study period; however, another study from Libya reported age-standardized incidence rates for HCC of 5.23 per 100,000 population and 5.88 per 100,000 population in 2017, and found that the main underlying disease contributing to HCC was HCV infection [23].

The incidence rate of different types of malignancies increases with advancing age; the majority of cancer cases (n= 284, 70.6%) were found in the older age groups of 41 years and above. This is consistent with the findings of other studies from Libya [7,9].

The histopathology records did not contain any data for lung cancer over the study period, although lung cancer was reported by other studies to be one of the most common cancers in Libyan men cancer patients [7,8,24]. The reason for this is that Tobruk Medical Centre lacks trained specialists and the proper equipment (e.g., lung endoscopy) to obtain biopsies from the lung; for this reason, patients with lung cancer are usually referred to other institutions (e.g., the Benghazi and Shahat thoracic clinics) to have histopathology samples taken.

**Figure 2.**
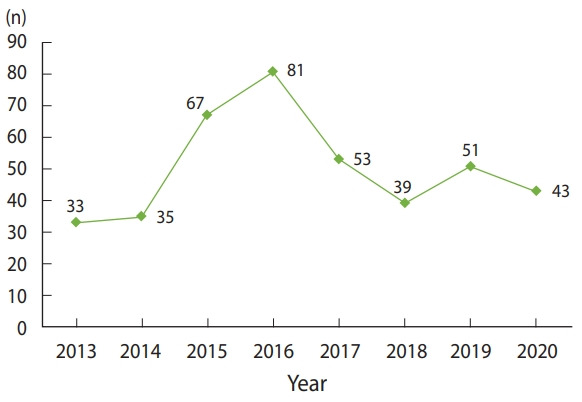
Malignancies incidence over the study period.

There was an increase in the number of diagnosed malignancies during the period from 2014 to 2017 (Figure 2). The main cause for this change was that the Department of Histopathology of Tobruk Medical Centre was the only functioning histopathology department in the region during the civil conflict in Benghazi and Derna cities that took place between 2014 and 2017.

Data on cancer incidence in Libya can contribute to a better understanding of the epidemiology of various cancers in the country, and therefore provide a useful guide for the health authorities to design and set into action a cancer prevention and control plan.

A major limitation of this study is that patients’ data lacked some important demographic and socioeconomic information, as well as data about the risk factors associated with cancer. Furthermore, data regarding lung cancer were unavailable due to a lack of the necessary equipment and trained personnel for collecting biopsies from the lung. For this reason, patients with lung tumours were usually referred to other institutions to have histopathology samples taken.

Furthermore, other limitations of data obtained from pathology-based cancer registries include missing cancer registrations or misclassifications of cancer sites, which may occur because of recording errors and may lead to overestimation or underestimation of some cancer types. In addition, under-registration of cancers diagnosed based on other diagnostic methods such as characteristics of radiological features or tumour markers (as may be relevant for cancers of the central nervous system, lung, prostate, and urinary tract, as well as HCC), may result in underestimation of the actual cancer incidence [25]. For example, HCC is diagnosed in patients with cirrhosis based on non-invasive criteria and/or pathology findings. Imaging is an essential part of the HCC diagnosis, contributing to the establishment of the type of primary liver tumour and staging of HCC. Non-invasive imaging for the diagnosis of HCC for patients with cirrhosis was accepted in 2001, when dynamic imaging explorations demonstrated the typical diagnostic pattern for HCC [26].

In conclusion, breast and uterine cancers were the most common cancers in women, and bladder and colorectal cancers were the most common in men, followed by colorectal cancer in both genders. This data will help the health authorities to start a prevention plan for cancer in the region. Further studies to identify etiological factors and cancer-related risk factors need to be conducted in the region.

## Figures and Tables

**Table 1.** The most common malignancies in the study population

Cancer type	Women	Men	Total, n (%)
Breast	74	0	74 (18.4)
Uterus	64	0	64 (15.9)
Colorectal	26	21	47 (11.6)
Bladder	8	25	33 (8.2)
Thyroid	23	9	32 (8.0)
Lymphoma	19	12	31 (7.7)
Skin melanoma	12	3	15 (3.7)
Sinonasal carcinoma	3	11	14 (3.5)
Oral cavity	4	7	11 (2.7)
Prostate	0	11	11 (2.7)
Kidney	7	2	9 (2.2)
Cervix	8	0	8 (2.0)
Gallbladder	7	1	8 (2.0)
Orofacial	5	3	8 (2.0)
Ovary	8	0	8 (2.0)
Nasopharynx	4	2	6 (1.5)
Small intestine	1	5	6 (1.5)
Bone	3	2	5 (1.2)
Stomach	3	2	5 (1.2)
Larynx	0	4	4 (1.0)
Brain	1	0	1 (0.2)
Oesophagus	0	1	1 (0.2)
Testis	0	1	1 (0.2)
Total	280	122	402 (100)
